# Modeling the spatio‑temporal spread of COVID‑19 cases, recoveries and deaths and effects of partial and full vaccination coverage in Canada

**DOI:** 10.1038/s41598-022-21369-z

**Published:** 2022-10-24

**Authors:** Somayeh Momenyan, Mahmoud Torabi

**Affiliations:** 1 Department of Community Health Sciences, Rady Faculty of Health Sciences, University of Manitoba, Winnipeg, MB R3E 0W3 Canada; 2 Department of Statistics, Faculty of Science, University of Manitoba, Winnipeg, Canada

**Keywords:** Diseases, Medical research, Risk factors

## Abstract

The purposes of our study are to map high-risk areas in Canada as well as quantifying the effects of vaccination intervention and socio-demographic factors on the transmission rates of infection, recovery, and death related to COVID-19. The data of this research included weekly number of COVID‑19 ﻿cases, recovered, and dead individuals from 2020 through 2021 in Canada at health region and provincial levels. These data were associated with cumulative rates of partial and full vaccination and socio-demographic factors. We applied the spatio-temporal Susceptible-Exposed-Infected-Removed (SEIR), and Susceptible-Exposed-Infected-Removed-Vaccinated (SEIRV) models. The results indicated the partial vaccination rate has a greater effect compared with full vaccination rate on decreasing the rate of infectious cases (risk ratio (RR) = 0.18; 95%CrI: 0.16–0.2; RR = 0.60; 95%CrI: 0.55–0.65, respectively) and increasing the rate of recovered cases (RR = 1.39; 95%CrI: 1.28–1.51; RR = 1.21; 95%CrI: 1.23–1.29, respectively). However, for mortality risk reduction, only increasing full vaccination rate was significantly associated (RR = 0.09; 95%CrI: 0.05–0.14). In addition, our results showed that regions with higher rates of elderly and aboriginal individuals, higher population density, and lower socioeconomic status (SES) contribute more to the risk of infection transmission. Rates of elderly and aboriginal individuals and SES of regions were significantly associated with recovery rate. However, elderly individuals rate of regions was only a significant predictor of mortality risk. Based on the results, protection against mild and severe COVID-19 infection after the primary vaccination series decreased.

## Introduction

COVID-19 outbreaks have spread globally since the beginning of 2020^1^. In Canada, since the identification of the first case in January 2020, a large number of cases and deaths related to COVID-19 have been reported^2^. Therefore, to curb this growing number of infections and deaths, several non-pharmaceutical and pharmaceutical interventions have been instituted by the Canadian government. More specifically, mandatory travel restrictions and physical distancing (including the closure of non-essential businesses, schools, and universities) were issued in mid-March 2020^3^. Also, the implementation of Pfizer-BioNTech and Moderna vaccines was authorized in December 2020^4^. Despite unprecedented interventions enacted in many countries, COVID-19 has caused severe global health challenges and tremendous economic concerns^5,6^. Although the transmission rate has slowed down after the implementation of these interventions, the outbreak pattern of COVID-19 still needs to be identified for implementing more intervention measures to eradicate the disease. Also, some serious issues have remained concerning the effect of vaccination intervention at the population level in real-world settings. Until the end of December 2021, about 57% of the world population has received at least one dose of a COVID-19 vaccine^7^. Hence, along with the improving rates of vaccination all over the world, there is an urgent need to understand the potential impact of vaccination on COVID-19 transmission dynamics.

Canada is going through the fifth wave at the time of this study, so it is still vital to understand the geographical evolution of COVID-19 for identifying regions with higher risk of infection transmission, severe infection, and mortality. Also, the effects of vaccination intervention and socio-demographic factors on transmission rates of infection, recovery, and mortality have not been fully evaluated in Canada. Therefore, the purposes of our study are to map high-risk areas in Canada as well as quantifying the effects of partial and full vaccination coverage at the population level and socio-demographic factors on transmission rates of infection, recovery, and death. Our results are expected to be useful for health care providers in Canada to prevent further spread of COVID-19.

## Methods

### Data collection

The data of this research were from Esri Canada’s COVID-19 dashboard^8^, which is collected from the Public Health Agency of Canada. The first dataset included weekly summaries on the number of cases by date of symptom onset, recovered, and dead individuals due to COVID-19 from April 5, 2020, through December 11, 2021 (88 weeks) in 92 health regions. The second dataset included the same weekly information from January 26, 2020, through December 11, 2021 (98 weeks) in 13 provinces. These data were associated with community information for each health region and province in Canada. The variables related to health regions and provinces which were obtained from the 2016 Canadian census included the rate of individuals aged over 65, the rate of aboriginal individuals, population density per square kilometer, and socioeconomic status (SES) containing information on median household income, proportion of individuals aged over 15 who have not graduated high school, and unemployment rate. A principal component analysis was performed for the calculation of the SES score. Also, we had variables of average household size and rate of individuals who work in health occupations which were not considered in our models as their variation between regions was trivial. So, the predictors of our study were similar to some previous literature^9–11^.

**Figure 1:**
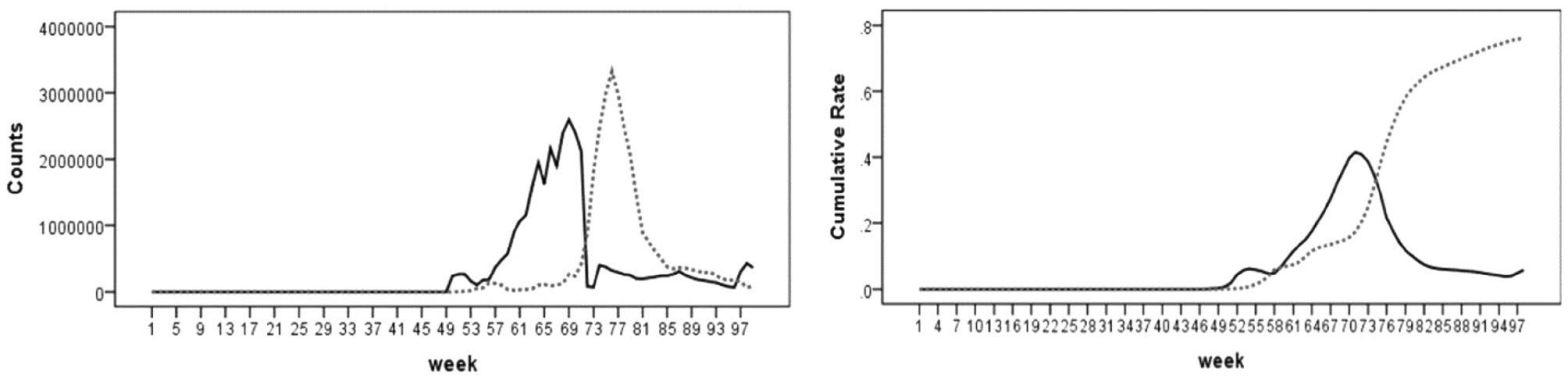
Counts of the first dose vaccination = solid line and the second dose vaccination = dotted line (left); cumulative rate of partial vaccination = solid line and full vaccination = dotted line (right) in provincial dataset.

In addition, information about the weekly cumulative rates of partial vaccination (those who have only received one dose of a two-dose course under a vaccine regimen) and full vaccination (those who have received the full series of a vaccine regimen) as well as the weekly number of individuals who have received the first and second doses of a vaccine was obtained from the Public Health Agency of Canada^12^. Apart from Janssen vaccine that requires a single dose, all other COVID-19 vaccines authorized for use in Canada require two doses to complete the primary vaccination series. Since only 0.06% of total population have received a single dose Janssen vaccine, we could consider the rate of partial and full vaccination as the rate of individuals who have received the first and second doses of a vaccine, respectively. Further information regarding COVID-19 vaccination data was shown in Fig. 1. As the left figure shows, there is a delaying interval between the implementation of the first and second vaccine doses in Canada. At the time of this analysis, 4% of total population has been partially vaccinated and 76% of total population has been fully vaccinated (the right figure). Since the vaccination data at health region level were incomplete, this information was only considered in the model for the provincial data to assess the impact of partial and full vaccination rates increase on infection, recovery, and death transmission rates.

### Model structure

**Figure 2:**
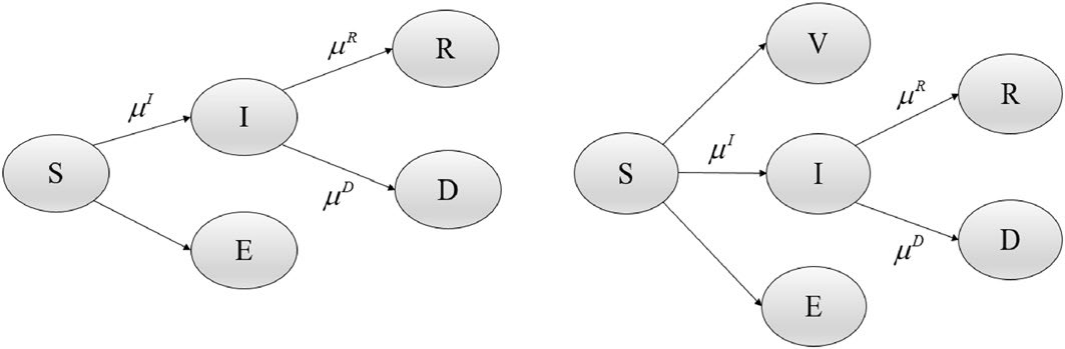
The SEIR (left) and SEIRV (right) models for the COVID-19 epidemic data. S: susceptible population, E: exposed or asymptomatic cases, I: symptomatic cases, R: recovered cases, D: dead cases and V: vaccinated cases. *μ*^*I*^, *μ*^*R*^ and *μ*^*D*^ are the transmission rates of infection, recovery, and death respectively.

We first applied the Bayesian spatio-temporal Susceptible-Exposed-Infected-Removed (SEIR) model proposed by^9^ to evaluate the spatio-temporal transmission of COVID-19 cases, recoveries, and deaths in health regions of Canada. For the SEIR model over spatial regions $\{ i = 1,\ldots,I\}$ and time points $\{ t_{j} :j = 1,\ldots,J\}$, let $\rm{S}_{ij}$ be susceptible population, $E_{ij}$ be exposed or asymptomatic cases, $\rm{I}_{ij}$ be symptomatic cases, $\rm{R}_{ij} \rm{ }$ be removed cases including recovered cases, $\rm{R}_{ij} \rm{ }$, and dead cases,$\rm{ D}_{ij}$ (Fig. 2). As we do not have information on asymptomatic cases, the asymptomatic cases are assumed a proportion of symptomatic cases as $\rm{E}_{ij - 1} = \varphi \rm{I}_{ij - 1}$ and also are not included in transmission infection rate﻿ (not yet infectious). Furthermore, we assume that total population size is fixed during the time of study. Thus, the susceptible population at region $i$ and week $j$ can be calculated as $S_{ij} = S_{ij - 1} - \rm{E}_{ij - 1} - \rm{I}_{ij - 1} - R_{ij - 1} - D_{ij - 1}$.

The second analysis for the provincial data is based on the SEIR epidemic model augmented by the vaccinated individuals leading to the Susceptible-Exposed-Infected-Removed-Vaccinated (SEIRV) epidemic model (Fig. 2). Our SEIRV epidemic model can only account for first or second dose vaccinated individuals, not both simultaneously. Therefore, the susceptible population at the region $i$ and week $j$ can be calculated as $S_{ij} = S_{ij - 1} - \rm{E}_{ij - 1} - \rm{I}_{ij - 1} - R_{ij - 1} - D_{ij - 1} - \gamma V_{ij - 3}^{1}$ or $S_{ij} = S_{ij - 1} - \rm{E}_{ij - 1} - \rm{I}_{ij - 1} - R_{ij - 1} - D_{ij - 1} - \gamma V_{ij - 2}^{2}$, in which $V^{1}$ and $V^{2}$ represent the individuals who have received the first and second doses, respectively. The parameter $\gamma$ is the rate of vaccinated individuals who become immune. For example, $\gamma = 0$ means the susceptible population has not decreased by individuals who have received vaccination. In other words, infection dynamics continue for vaccinated individuals. Also, $\gamma = 1$ means all vaccinated individuals are immune, and they are no longer included in the susceptible population during the time of study. Since significant protective effect starts to develop at least 14 days after the first dose and 7 days after the second dose^13,14^, susceptible population is assumed to decrease by vaccinated individuals who have received the first or second dose in three and two previous weeks, respectively.

In both models, it is reasonable to assume a Poisson model for symptomatic, recovered, and dead cases at region $i$ and week $j$ as follows:

$$ \begin{gathered} I_{ij} \sim Pois\left( {\mu_{ij}^{I} } \right), \hfill \\ R_{ij} \sim Pois\left( {\mu_{ij}^{R} } \right), \hfill \\ \rm{D}_{ij} \sim Pois\left( {\mu_{ij}^{D} } \right). \hfill \\ \end{gathered} $$

The transmission rates of infection, recovery, and death are modeled to account for the transmission of pathogen, potential spatial and temporal correlation in data, effects of cumulative rates of partial and full vaccination (provincial data), and socio-demographic factors as follows:

$$ \begin{aligned} \log \left( {\mu_{ij}^{I} } \right) & = \log (S_{ij} ) + \log (\rm{I}_{ij - 1} ) + b_{0}^{I} {\mathbf{ + x}}_{ij}^{T} {{\varvec{\upbeta}}} + u_{i}^{I} + v_{i}^{I} , \\ \log \left( {\mu_{ij}^{R} } \right) & = \log (\rm{I}_{ij - 1} ) + b_{0}^{R} {\mathbf{ + x}}_{ij}^{T} {{\varvec{\upbeta}}} + u_{i}^{R} + v_{i}^{R} , \\ \log \left( {\mu_{ij}^{D} } \right) & = \log (\rm{I}_{ij - 2} ) + b_{0}^{D} {\mathbf{ + x}}_{ij}^{T} {{\varvec{\upbeta}}} + u_{i}^{D} + v_{i}^{D} . \\ \end{aligned} $$

In the above models, ${\varvec{b}} = \left( {b_{0}^{I} ,b_{0}^{R} ,b_{0}^{D} } \right)$ are intercept coefficients that are log-transformed baseline transmission rate for infection, recovery, and death, respectively across all regions. Also, ${{\varvec{\upbeta}}}$ is a vector of regression parameters associated with observed explanatory covariates. Let ${\varvec{u}}^{I} ,{\varvec{u}}^{R} \rm{ and }{\varvec{u}}^{D}$ be the spatial random effects and ${\varvec{v}}^{I} ,{\varvec{v}}^{R} \rm{ and }{\varvec{v}}^{D}$ be the unstructured random effects.

In this study, a variety forms of transmission rate for infection, recovery, and death were assessed in the COVID-19 data for both SEIR and SEIRV models. Transmission rates with no random effects, spatial random effects, unstructured random effects, and both spatial and unstructured random effects were examined. In addition, the best model according to the type of random effects was evaluated along with time-dependent intercept coefficients, ${\varvec{b}} = \left( {b_{0j}^{I} ,b_{0j}^{R} ,b_{0j}^{D} } \right)$. For the comparison between these alternative models, two measures were selected as follows: the deviance information criterion (DIC)^15^ and the mean squared prediction error (MSPE)^16^. The DIC criterion is based on the posterior distribution of deviance statistic and an effective number of parameters. MSPE is a Bayesian version of the mean squared error and measures the mean squared distance between the observed outcomes with their fitted values. The model with the smallest of DIC and MSPE among a collection of models is the preferred model.

### Model implementation

The Bayesian approach was considered for parameter estimation of model. Prior distributions were chosen to be as non-informative as possible. For regression parameters, the standard normal prior was used. For spatial and unstructured random effects an intrinsic conditional autoregressive (ICAR) and an uncorrelated zero mean normal prior were used, respectively. For time-dependent intercept coefficients, a zero mean normal prior with precision having a gamma distribution was considered. For the asymptomatic proportion parameter, three numbers of 0.1, 0.25, and 0.5 were examined in data suggested by^11^. For the immunity rate parameter, the numbers between 0 and 1 with a spacing of 0.1 were examined for both doses. Finally, for each parameter, the mean point estimate and standard deviation were calculated. In addition, for regression coefficients, adjusted risk ratio (RR) was estimated with a 95% credible interval. All analyses were performed using R package R2OpenBUGS. All methods were carried out in accordance with the relevant guidelines and regulations.

## Results

Daily data were aggregated on a weekly basis (from Sunday to Saturday) in health regions and provincial datasets to take into account the anomalies, such as weekend reporting delays. We analyzed both datasets using five proposed models with different forms of transmission rate. The models were fitted based on sampling chains of 50,000 iterations with the first 5000 discarded as a burn-in. Table 1 compared proposed models based on two criteria, DIC, and MSPE in two datasets. Based on the DIC and MSPE values, the second model which considers spatial random effects had a better fit in describing data among the first four models. In addition, the fifth model, which incorporates spatial random effects as well as time-dependent intercept coefficient in transmission rate, showed better comparison measures values for both datasets. Also, for all models, the asymptomatic rate assumption of 0.5 yielded a better goodness of fit result than other values for both datasets (not shown). This means for every two symptomatic cases there is likely to be one asymptomatic case. For provincial data, all five SEIRV models were checked by using the first or second dose vaccinated individuals for calculating susceptible population and values between 0 and 1 for immunity rate. Results showed using the first dose vaccinated individuals along with the immunity rate of 0.5 in all models has a better goodness of fit result (not shown). In other words, the model that the susceptible population has decreased by half of the individuals who have received the first dose of vaccination has a better fit than other models. It is worth mentioning that in all SEIRV models instead of predictors of partial and full vaccination cumulative rates at week $j$ the corresponding rates at three and two previous weeks, respectively were examined had a worse fit.

**Table 1:** Models comparison results, bold number shows the best fit.

	Random effects	Health regions data (SEIR model)		Provincial data (SEIRV model)	
		DIC	MSPE	DIC	MSPE
Model 1	No random effects	1,134,000	47,390	621,899	546,700
Model 2	Spatial random effects	587,600	18,370	527,520	531,900
Model 3	Unstructured random effects	587,700	18,380	527,545	532,300
Model 4	Spatial & unstructured random effects	587,620	18,370	527,522	532,300
Model 5	Spatial random effects & Time-dependent intercept	**464,800**	**11,540**	**327,910**	**283,700**

The parameters estimates of the fifth model for both datasets were presented in Table 2. Based on the adjusted risk ratio estimates in provincial data, there were significant relationships between partial and full vaccination coverage rates at population level with infection transmission rate. The results indicated that increase in cumulative rates of the first and second vaccine doses decreases the log expected rate of infectious cases. That is, the expected rate of infectious cases with one unit increase in the rate of the first dose is estimated to decrease by 82% (RR = 0.18; 95%CrI: 0.16–0.21) while with a one unit increase in the rate of second dose it is estimated to decrease by 40% (RR = 0.60; 95%CrI: 0.55–0.65). The adjusted relationships between partial and full vaccination coverage rates with recovery transmission rate were significant. Specifically, each unit increase in the rate of first and second vaccine doses yields an increase of 39% (RR = 1.39; 95%CrI: 1.28–1.51) and 21% (RR = 1.21; 95%CrI: 1.23–1.29) in the estimated rate of recovered cases, respectively. Also, our results showed with only increase of full vaccination coverage rate at population level the risk of mortality significantly decreases. So, each unit of increase in the rate of the second vaccine dose has a 0.91% (RR = 0.09; 95%CrI: 0.05–0.14) reduction in the expected rate of mortality.

**Table 2:** Estimation results of the best models for the COVD-19 data.

Parameters	Health regions data (SEIR model)		Provincial data (SEIRV model)	
	Mean (SD)	Risk ratio (95% credible interval)	Mean (SD)	Risk ratio (95% credible interval)
**Infection rate **				
Partial vaccination coverage	–	–	− 1.681 (0.054)	**0.186 (0.164, 0.212)**
Full vaccination coverage	–	–	− 0.499 (0.052)	**0.607 (0.554, 0.659)**
Rate of individuals aged over 65	0.148 (0.010)	**1.160 (1.156, 1.179)**	0.119 (0.005)	**1.126 (1.111, 1.148)**
Rate of aboriginal individuals	0.039 (0.014)	**1.041 (1.019, 1.046)**	0.103 (0.003)	**1.109 (1.093, 1.120)**
SES score	− 0.629 (0.125)	**0.537 (0.492, 0.532)**	− 0.613 (0.305)	**0.566 (0.399, 0.890)**
Population density per km^2^	0.087 (0.012)	**1.092 (1.071, 1.107)**	0.096 (0.008)	**1.101 (1.086, 1.115)**
$\sigma_{{b_{0} }}^{2}$: Time-dependent intercept	0.070 (0.010)	–	0.337 (0.052)	–
$\sigma_{u}^{2}$: Spatial random effects	4.030 (0.825)	–	5.986 (2.809)	–
**Recovery rate**				
Partial vaccination coverage	–	–	0.329 (0.046)	**1.391 (1.282, 1.514)**
Full vaccination coverage	–	–	0.192 (0.044)	**1.213 (1.123, 1.294)**
Rate of individuals aged over 65	0.111 (0.006)	**1.117 (1.100, 1.133)**	0.079 (0.011)	**1.083 (1.066, 1.111)**
Rate of aboriginal individuals	0.038 (0.019)	**1.040 (1.014, 1.050)**	0.102 (0.008)	**1.108 (1.090, 1.127)**
SES score	1.003 (0.275)	**2.829 (1.845, 2.683)**	0.749 (0.295)	**2.206 (1.325, 2.331)**
$\sigma_{{b_{0} }}^{2}$: Time-dependent intercept	0.049 (0.007)	–	0.215 (0.036)	–
$\sigma_{u}^{2}$: Spatial random effects	2.676 (0.674)	–	6.135 (4.663)	–
**Death rate**				
Partial vaccination coverage	–	–	− 0.107 (0.276)	0.933 (0.528, 1.487)
Full vaccination coverage	–	–	− 2.343 (0.256)	**0.099 (0.059, 0.147)**
Rate of individuals aged over 65	0.054 (0.019)	**1.056 (1.031, 1.061)**	0.114 (0.020)	**1.122 (1.087, 1.166)**
Rate of aboriginal individuals	0.005 (0.006)	1.005 (0.984, 1.017)	0.021 (0.014)	1.022 (0.989, 1.051)
SES score	− 0.093 (0.071)	0.912 (0.805, 1.041)	− 0.328 (0.371)	0.734 (0.342, 1.324)
$\sigma_{{b_{0} }}^{2}$: Time-dependent intercept	0.477 (0.074)	–	0.785 (0.130)	–
$\sigma_{u}^{2}$: Spatial random effects	0.488 (0.115)	–	0.559 (0.384)	–

Socioeconomic status (SES) score: Increasing score indicates a higher level of socioeconomic status.

Significant results were shown in bold.

﻿In addition, there were significant relationships between the rate of individuals aged over 65, the rate of aboriginal individuals, population density, and SES with infection transmission rate in two datasets. In other words, regions with a higher rate of elderly and aboriginal individuals, higher population density, and lower SES are estimated to have a higher rate of infectious cases compared to other regions. Based on the results of both datasets, higher rate of elderly and aboriginal individuals and SES in regions appeared to have significant associations with higher rate of recovered cases. However, the rate of elderly individuals in regions was only a significant predictor of mortality risk in both datasets.

**Figure 3:**
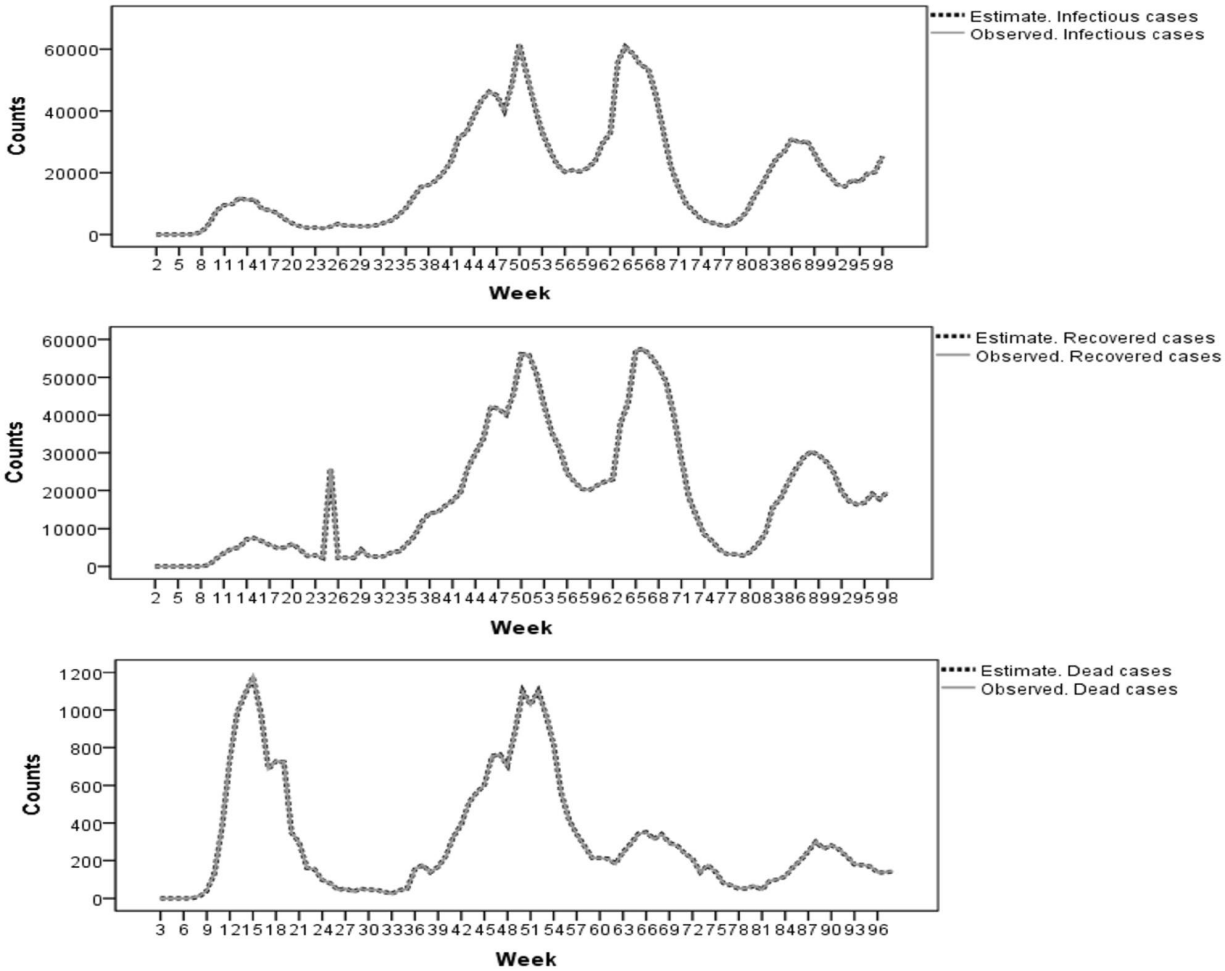
Observed and fitted number of infectious, recovered, and dead cases based on the SEIRV model in provincial dataset.

The posterior mean estimates of infection, recovery, and death temporal profiles across all provinces based on provincial dataset were displayed in Fig. 3. These profiles demonstrate that the fifth model fitted the observed infectious, recovered, and dead case count profiles very well, so that the observed and the fitted values had very small differences. Also, the observed case count profiles of the infectious, recovered, and dead based on health regions dataset were fitted similarly very well (not shown).

**Figure 4:**
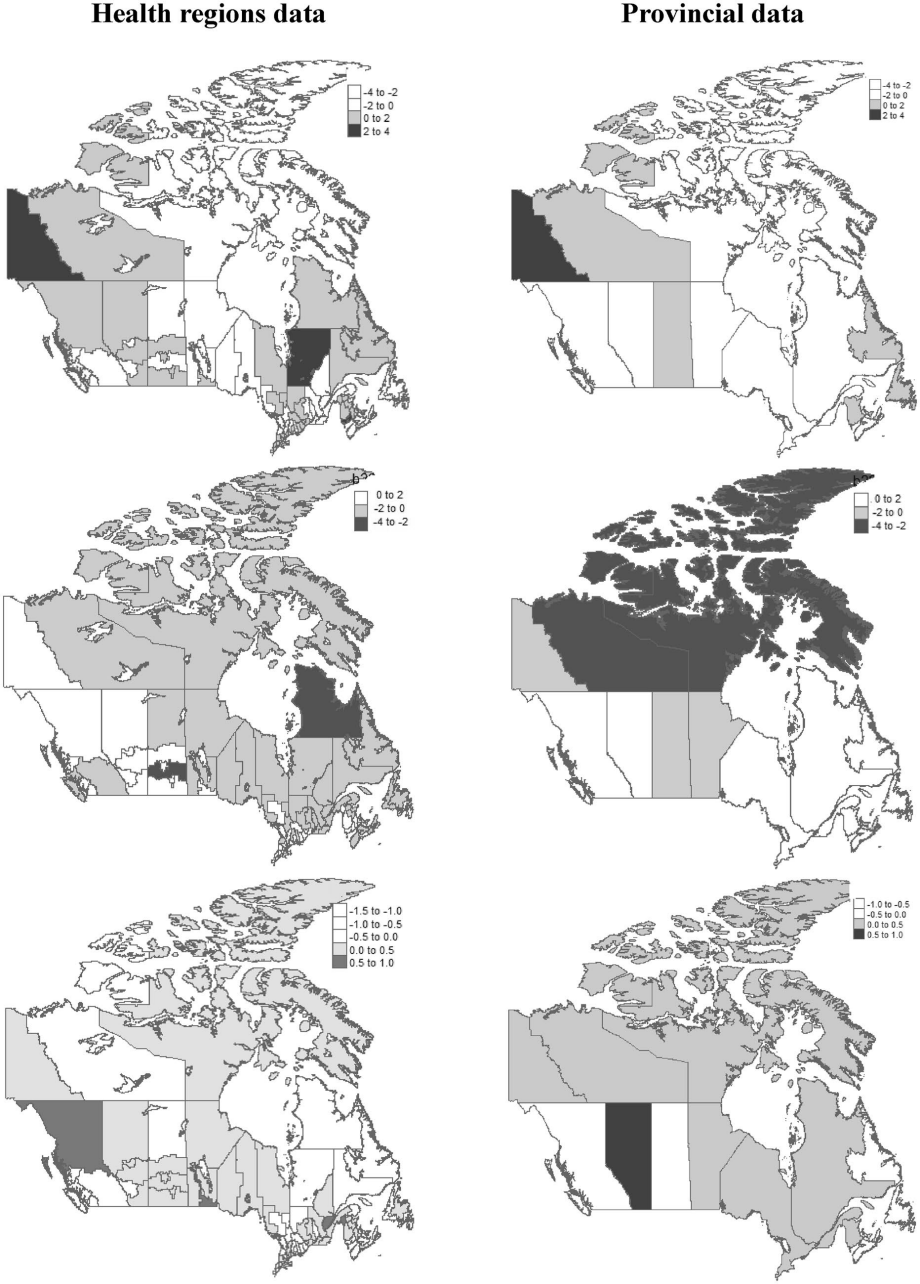
The posterior mean of spatial random effects related to the infection, recovery, and death transmission rates, in upper, middle, and bottom plots, respectively. In all maps, the posterior estimates of random effects were recorded based on the percentiles of their distribution to show spatial inequalities. Maps constructed using R-4.2.1 (https://www.r-project.org).

Finally, spatial random effects estimate of the fifth model was mapped for both datasets (Fig. 4). As shown in the upper figures, four health regions (Northern Quebec, Yukon, Saint John, Northwest Territories) and one province (Yukon) were identified with the higher risk of infection transmission. Also, three health regions (Nunavik and Terres-Cries-de-la-Baie-James regions in Quebec, Central region in Saskatchewan) and two provinces (Northwest Territories, Nunavut) were identified with the lower recovery transmission rate or higher severe infection (middle figures). For the risk of mortality, five health regions (Southern Health and Winnipeg in Manitoba, Edmundston in New Brunswick, Capitale-Nationale in Quebec, Northern Health in British Columbia) and one province (Alberta) were identified with the higher risk (bottom figures).

## Discussion

In order to help to the surveillance programs centers, evaluating dynamics of COVID-19 cases, recovered and dead people over time as well as assessing the impact of risk factors to the differences in risk over space and time are needed. In this study, we have demonstrated the use of the Bayesian spatio-temporal SEIR and SEIRV models for the estimation of infection, recovery, and death transmission rates of the COVID-19 outbreak in Canada at health region and provincial levels. In our datasets, the SEIR and SEIRV models, which incorporate spatial random effects, time-dependent intercept coefficient as well as other predictors in transmission rates yielded better and more reasonable measures of fit. This suggests that baseline transmission rates were heterogeneous during the time of COVID-19 outbreak in Canada. Also, the asymptomatic rate of 50% and the immunity rate of 50% had a better fit than other values. Our asymptomatic rate was similar to the rates previously reported^11,17^.

We considered the potential confounders in the model to enable adjusted analysis for vaccination effect. Our results showed that increasing first dose of vaccination rate at population level has a greater effect compared with second dose of vaccination rate on decreasing the rate of infectious cases and increasing the rate of recovered cases. The second dose of vaccination, however, was more effective than first dose of vaccination on reducing the risk of mortality. Also, first and second doses of vaccination had the greatest effect on infectious and dead cases rates, respectively. Diminished efficacy of the second dose in infection and recovery transmission rates in our findings may be due to several reasons. First, the delayed second dose strategy instead of adhering to the dosing strategy tested in the clinical trials. This gap was the result of the Canadian government’s decision to focus on giving the first dose to as many people as possible to protect them from severe infection and death. However, some studies have shown that delayed dosing can result in a stronger immune response for some subgroups^18–20^. Second, protection against different variants may be decreased over time after receiving the second dose. This reason is consistent with recent findings from some studies that showed waning of protection over time after receiving the second dose against infection^21–24^ and hospitalization^21,23^. There may be an extra reason for infection transmission rate that is related to people being less cautious about protective behaviors. It is due to the fact that they think receiving second dose of vaccination brings about full immunity and also Canada was relaxing restrictive measures, such as social distancing along with the improvement rate of second vaccine dose. Furthermore, similar to the earlier studies, we found with completion of a primary vaccine series the protection against death increases^21,25–27^. Finally, it is clear from our results that protection against mild and severe COVID-19 infection after the primary vaccination series decreased. Therefore, if we have a plan to reduce the magnitude of subsequent waves of COVID-19 in the presence of new variants, the protection should be enhanced with increasing rate of booster dose at population level. More recent findings indicated that the third dose was significantly associated with large reductions in the incidence of infectious cases^28^.

Some of the spatio-temporal differentiations of the COVID-19 epidemic across Canada could be explained by population characteristics. For example, based on our results, we might expect regions with higher elderly and aboriginal people rates, higher population density, and lower SES contribute more to the risk of infection transmission which is in line with previous studies^9,11,29^. Also, the regions with the high proportion of elderly population were significantly at a higher risk of COVID-19 related mortality which is consistent with the results of earlier reports^9,30,31^.

The inclusion of spatial random effects allows for residual confounding factors in transmission rate that are spatially structured. In other words, the values of spatial random effects can be interpreted as a residual risk after adjusting for covariates effects. So, the observed results in maps do not correlate with socio-demographic differences between regions which were considered in the model. As all maps showed most regions with the greatest residual risk are rural in nature that efficient surveillance programs should be ensured in these regions. Also, the urban regions had lower residual risk.

There were some limitations in our available datasets that could lead to biased estimates. First, the asymptomatic and symptomatic patients who did not attend COVID-19 screening tests were not counted. Second, the asymptomatic recovery and death cases were not included as they were unmeasured. Third, our model did not consider the age-structured transmission rates because the age variable was not available in our datasets. Fourth, the information on the proportion of second dose vaccinated individuals who received the first dose was not available in the provincial dataset, that is why our model could not account for multiple vaccinations, such as the Susceptible-Exposed-Infected-Removed-Vaccinated1 (first dose)-Vaccinated2 (second dose) (SEIRVV) model^32^.

## Supplementary Information

Supplementary Information.

## Data Availability

The codes that support the finding of this study are available within the Supplementary Information.
