# Supplementary material for: Modeling the spatio‑temporal spread of COVID‑19 cases, recoveries and deaths and effects of partial and full vaccination coverage in Canada
Source: Sci Rep. 2022 Oct 24;12:17817. doi: 10.1038/s41598-022-21369-z (PMC9589715; doi:10.1038/s41598-022-21369-z)
Supplement: Supplementary file 1 — Supplementary Information. [file 41598_2022_21369_MOESM1_ESM.docx]

**Appendix**

**The code for the model 5**

-----------------------------------------------------------------------------------

i: spatial region

j: time

I: infectious cases

R: recovered cases

D: dead cases

Susc: susceptible population which is made in the dataset based on equations mentioned in the paper

**Model 5:**

model{

for (i in 1:K){

I[i,1]~dpois(muc[i,1])

muc[i,1]<-0.001*susc[i,1]

R[i,1]~dpois(mur[i,1])

mur[i,1]<-0.1*I[i,1]

D[i,1]~dpois(mud[i,1])

mud[i,1]<-0.01*I[i,1]

}

for (i in 1:K){

I[i,2]~dpois(muc[i,2])

log(muc[i,2])<-log(susc[i,2]+0.001)+log(I[i,1]+0.001)+b1[i]+bet10[2]+bet11*x11[i,2]+bet12*x22[i,2]+bet13*x33[i]+bet14*x44[i]+bet15*x55[i]+bet16*x66[i]

R[i,2]~dpois(mur[i,2])

log(mur[i,2])<-log(I[i,1]+0.001)+b2[i]+bet20[2]+ bet21*x11[i,2]+ bet22*x22[i,2]+bet23*x33[i]+bet24*x44[i]+bet25*x55[i]

D[i,2]~dpois(mud[i,2])

mud[i,2]<-0.01*I[i,2]

}

for (i in 1:K){

for (j in 3:T){

I[i,j]~dpois(muc[i,j])

log(muc[i,j])<-log(susc[i,j]+0.001)+log(I[i,j-1]+0.001)+b1[i]+bet10[j]+bet11*x11[i,j]+bet12*x22[i,j]+bet13*x33[i]+bet14*x44[i]+bet15*x55[i]+bet16*x66[i]

R[i,j]~dpois(mur[i,j])

log(mur[i,j])<-log(I[i,j-1]+0.001)+b2[i]+bet20[j]+ bet21*x11[i,j]+ bet22*x22[i,j]+bet23*x33[i]+bet24*x44[i]+bet25*x55[i]

D[i,j]~dpois(mud[i,j])

log(mud[i,j])<-log(I[i,j-2]+0.001)+b3[i]+bet30[j]+bet31*x11[i,j]+bet32*x22[i,j]+bet33*x33[i]+bet34*x44[i]+bet35*x55[i]

}

}

b1[1:13]~ car.normal(adj[], weights[], num[], tau.b1)

b2[1:13]~ car.normal(adj[], weights[], num[], tau.b2)

b3[1:13]~ car.normal(adj[], weights[], num[], tau.b3)

for(i in 1:38) {weights[i] <- 1}

tau.b1~dgamma(0.5, 0.0005)

sigma1<-(1/tau.b1)

tau.b2~dgamma(0.5, 0.0005)

sigma2<-(1/tau.b2)

tau.b3~dgamma(0.5, 0.0005)

sigma3<-(1/tau.b3)

bet11~dnorm(0,1)

bet12~dnorm(0,1)

bet13~dnorm(0,1)

bet14~dnorm(0,1)

bet15~dnorm(0,1)

bet16~dnorm(0,1)

bet21~dnorm(0,1)

bet22~dnorm(0,1)

bet23~dnorm(0,1)

bet24~dnorm(0,1)

bet25~dnorm(0,1)

bet31~dnorm(0,1)

bet32~dnorm(0,1)

bet33~dnorm(0,1)

bet34~dnorm(0,1)

bet35~dnorm(0,1)

for (k in 2:T){

bet10[k]~dnorm(alpha10,tau10)}

tau10~dgamma(2, 0.5)

sigma10<-(1/tau10)

alpha10~dflat()

for (k in 2:T){

bet20[k]~dnorm(alpha20,tau20)}

tau20~dgamma(2, 0.5)

sigma20<-(1/tau20)

alpha20~dflat()

for (k in 3:T){

bet30[k]~dnorm(alpha30,tau30)}

tau30~dgamma(2, 0.5)

sigma30<-(1/tau30)

alpha30~dflat()

}
